# Metabolic Dysfunction–Associated Steatotic Liver Disease as a Systemic Driver of Heart Failure With Preserved Ejection Fraction: Mechanistic Insights and Emerging Therapeutic Perspectives

**DOI:** 10.31083/RCM51234

**Published:** 2026-06-26

**Authors:** Junli Wu, Wenwen Zheng, Qianxian Qi, Chaojie He, Changlin Zhai, Hongyan Fan

**Affiliations:** ^1^Department of Endocrinology, The Second Affiliated Hospital of Jiaxing University, 314001 Jiaxing, Zhejiang, China; ^2^Department of Cardiology, The Affiliated Hospital of Jiaxing University, 314001 Jiaxing, Zhejiang, China

**Keywords:** metabolic dysfunction–associated steatotic liver disease, heart failure with preserved ejection fraction, metabolic dysfunction, pathophysiology, therapeutic approaches

## Abstract

Heart failure with preserved ejection fraction (HFpEF) has emerged as the predominantform of heart failure (HF) worldwide and is increasingly recognized as a systemic syndrome closely linked to metabolic dysfunction. Furthermore, metabolic dysfunction–associated steatotic liver disease (MASLD), a highly prevalent yet often underrecognized comorbidity in patients with HFpEF, has attracted increasing attention. Accumulating epidemiological evidence demonstrates a significant association between MASLD and HFpEF, suggesting shared pathophysiological foundations that extend beyond coincidental coexistence. Mechanistically, MASLD contributes to the development and progression of HFpEF through a network of interconnected pathways, including chronic low-grade inflammation, insulin resistance (IR), dysregulated lipid metabolism, endothelial dysfunction, the gut–liver–heart axis, and as liver-derived mediators that influence cardiac structure and function. These overlapping mechanisms underlie the pronounced clinical and phenotypic heterogeneity of HFpEF and may help explain the limited efficacy of conventional heart failure therapies. This review summarizes current diagnostic and therapeutic strategies for HFpEF and MASLD and proposes an integrated, multiorgan framework to improve clinical recognition and management. A deeper understanding of liver–heart interactions is essential to redefining cardiometabolic disease, shifting from an organ-centric perspective toward a more integrated, mechanism-based approach.

## 1. Introduction

Metabolic dysfunction–associated steatotic liver disease (MASLD) represents a paradigm shift in the conceptualization of fatty liver disease, emphasizing metabolic dysfunction as the primary driver of hepatic steatosis rather than defining the condition by the exclusion of secondary causes [1,2]. Beyond hepatic involvement, MASLD is strongly associated with obesity, type 2 diabetes mellitus (T2DM), and dyslipidemia, and is now recognized as an independent and modifiable contributor to cardiovascular disease (CVD) risk [3,4,5].

Accumulating evidence supports a tightly interconnected and bidirectional relationship between MASLD and CVD. Shared pathophysiological mechanisms—including endothelial dysfunction and chronic low-grade inflammation—concurrently drive hepatic injury and cardiovascular remodeling. These processes promote not only atherosclerosis and vascular dysfunction but also myocardial structural and functional alterations [6,7]. Notably, emerging data indicate that liver fibrosis stage, rather than steatosis burden or obesity per se, is the strongest determinant of cardiovascular outcomes, with fibrosis closely correlating with subclinical atherosclerosis and cardiovascular mortality [6,7]. This framework also helps explain why individuals with lean MASLD may exhibit substantial cardiometabolic risk despite the absence of overt obesity.

Heart failure with preserved ejection fraction (HFpEF), a prevalent cardiovascular phenotype among patients with MASLD, is increasingly recognized as a systemic cardiometabolic syndrome. It is characterized by impaired diastolic function, increased myocardial stiffness, and a high burden of metabolic comorbidities [8]. Rather than representing a discrete entity, HFpEF lies along a continuum of left ventricular ejection fraction (LVEF), with heart failure with mildly reduced ejection fraction (HFmrEF) representing an intermediate phenotype that shares structural and mechanistic features with both HFpEF and heart failure with reduced ejection fraction (HFrEF) [9]. In the context of MASLD, subclinical myocardial dysfunction—including mildly reduced ejection fraction and impaired myocardial relaxation—has been increasingly reported, likely reflecting shared mechanisms such as myocardial fibrosis, systemic inflammation, and microvascular dysfunction [10].

Within this evolving framework, MASLD is increasingly viewed not merely as a comorbidity but as a systemic driver of HFpEF. Through integrated pathways involving inflammation, insulin resistance (IR), lipotoxicity, endothelial dysfunction, and inter-organ crosstalk, MASLD contributes to myocardial remodeling, ventricular stiffening, and impaired cardiac reserve. This integrative perspective provides a mechanistic basis for the heterogeneity and therapeutic challenges of HFpEF and supports the concept of a unified cardiometabolic axis linking liver and heart disease.

This narrative review synthesizes current evidence on the epidemiology, pathophysiological mechanisms, and therapeutic implications of MASLD in HFpEF. Relevant literature was identified through systematic searches of PubMed, Embase, and Web of Science, with emphasis on recent high-quality clinical and mechanistic studies. By integrating insights from hepatology, cardiology, and metabolic science, this review aims to provide a comprehensive framework to inform future research and enable precision therapeutic strategies.

## 2. HFpEF as a Metabolically Heterogeneous Syndrome

HFpEF is increasingly being recognized as a systemic syndrome characterized by marked clinical and biological heterogeneity rather than as a single cardiac disorder. Although elevated left ventricular filling pressures and diastolic dysfunction remain core diagnostic features, HFpEF encompasses a wide range of pathophysiological processes that extend beyond the myocardium [11]. More importantly, HFpEF is now widely regarded as a metabolically heterogeneous syndrome that is influenced to varying degrees by distinct patterns of metabolic dysfunction, including obesity, insulin resistance, T2DM, and ectopic fat deposition [12,13,14]. These metabolic perturbations interact with chronic low-grade inflammation, endothelial and microvascular dysfunction, impaired myocardial energetic flexibility, and extracardiac organ involvement, collectively shaping the diverse clinical manifestations of HFpEF.

Recognition of HFpEF heterogeneity has driven efforts to classify patients on the basis of clinically and biologically meaningful phenotypes. Among these, metabolic phenotypes—particularly those associated with obesity and diabetes—have emerged as dominant and highly prevalent subgroups. Obese HFpEF is characterized by plasma volume expansion, systemic inflammation, increased epicardial and visceral adiposity, and a disproportionate symptom burden despite relatively modest structural cardiac abnormalities [15,16]. Similarly, diabetic HFpEF is characterized by insulin resistance, altered myocardial energy metabolism, microvascular dysfunction, and a disproportionately high risk of adverse clinical outcomes [13]. Importantly, these metabolic phenotypes are not solely defined by the presence of comorbidities but reflect shared underlying pathophysiological mechanisms that shape disease progression and therapeutic responsiveness.

Within this framework, MASLD is a critical yet underexplored component of the metabolic spectrum of HFpEF. MASLD is characterized by hepatic steatosis in the context of systemic metabolic dysfunction, encompassing multiple cardiometabolic abnormalities that are mechanistically linked to HFpEF pathogenesis [17,18]. Given the central role of the liver in regulating lipid metabolism, glucose homeostasis, inflammatory signaling, and interorgan endocrine communication, MASLD can be viewed as a concentrated hepatic manifestation and a key driver of systemic energy metabolic dysregulation [19]. Accordingly, examining HFpEF through the lens of MASLD may provide novel insights into the disease mechanisms, phenotypic stratification, and opportunities for targeted therapeutic interventions.

## 3. Epidemiological Association Between MASLD and HFpEF

As the prevalence of metabolic disorders continues to rise, HFpEF and MASLD have emerged as the predominant form of heart failure (HF) and the most common chronic liver disease, respectively. Since the late 20th century, HFpEF has been the principal driver of the increasing global HF burden, accounting for more than 50% of newly diagnosed HF cases, with this proportion continuing to grow [20]. Among patients with HFpEF, up to 80% are overweight or obese, and 40%–50% have concomitant T2DM [21,22]. Similarly, MASLD affects approximately 30% of the global adult population; its prevalence rises to 55%–70% in individuals with T2DM and 70%–75% in those who are overweight or obese [8,23,24,25]. Notably, metabolic syndrome represents a strong predictor for both HFpEF and MASLD [26]. In addition, large-scale community-based cohort studies have demonstrated that MASLD remains an independent risk factor for incident HF and HF-related mortality, even after adjustment for traditional cardiometabolic risk factors commonly associated with metabolic syndrome [27,28,29,30]. Furthermore, the severity of MASLD is independently associated with an increased risk of incident HF, worse clinical outcomes, and higher HF risk scores [31,32].

Substantial epidemiological evidence indicates a close association between MASLD and the development and progression of HF, with a particularly pronounced link to HFpEF. A study involving 870,535 Medicare beneficiaries (including 27,919 with a clinical diagnosis of MASLD) demonstrated that MASLD was independently associated with the risk of HF, and its association with incident HFpEF (adjusted hazard ratio [aHR] 1.24, 95% confidence interval [CI] 1.14–1.34) was notably stronger than that with HFrEF (aHR 1.09, 95% CI 0.98–1.20) [33]. This predilection for HFpEF was further supported by another large retrospective cohort study. Among the 26,676 included patients with steatotic liver disease (SLD), approximately 70% had MASLD. During a median follow-up of 6 years, patients with MASLD exhibited a significantly increased risk of incident HF, with 76% of the new HF events being HFpEF. After multivariable adjustment, MASLD remained specifically and significantly associated with HFpEF (subdistribution hazard ratio [sHR] 1.91, 95% CI 1.27–2.86) [34]. Notably, the results of this study also suggest that the cumulative burden of cardiometabolic risk factors might influence the relationship between MASLD and HFpEF—the risk of HF increased in a dose-dependent manner with a greater number of metabolic risk factors, highlighting the interplay between MASLD and systemic metabolic dysfunction in terms of jointly driving HF risk [35]. Furthermore, research on related phenotypes, such as disproportionate liver fat (DLF), also supports the link between abnormal hepatic fat levels and HFpEF risk. In the Multi-Ethnic Study of Atherosclerosis, individuals in the highest DLF quartile more frequently exhibited adverse metabolic features, including dysglycemia and elevated triglyceride levels, which are associated with adverse cardiac remodeling and an increased risk of HFpEF [35].

The impact of MASLD on HFpEF extends beyond the disease onset phase, as its prognostic significance in patients with established HFpEF is also considerable. An analysis by Minhas et al. [36] based on the 2016–2018 U.S. National Inpatient Sample database revealed that among 1,361,740 hospitalized HFpEF patients, 2.4% had comorbid MASLD. Compared with HFpEF patients without MASLD, those with MASLD had significantly higher in-hospital mortality (adjusted odds ratio [aOR] 1.65, 95% CI 1.43–1.90, *p* < 0.001), along with significantly higher rates of adverse clinical events (including vasopressor use, cardiogenic shock, cardiac arrest, acute kidney injury, and progression to requiring dialysis). The length of hospital stay and healthcare costs increased correspondingly. Furthermore, the presence and severity of liver fibrosis in MASLD are also closely associated with HFpEF prognosis. In particular, advanced fibrosis (F3–F4) is strongly linked to adverse cardiovascular outcomes [37]. MASLD patients with advanced fibrosis exhibit more pronounced diastolic dysfunction and increased left atrial diameter [38]. Notably, this association persists even in non-obese individuals [39]. Collectively, these findings establish MASLD as a significant and fibrosis-modulated determinant of HFpEF risk and prognosis, highlighting a strong epidemiological and clinical link between the two conditions. These findings suggest that MASLD and HFpEF are linked beyond simple coexistence, underscoring the need to elucidate their shared pathophysiological mechanisms.

Epidemiological evidence on the association between MASLD and HFpEF is summarized in Table 1 (Ref. [33,34,35,36,37,38,39]).

**Table 1.** **Epidemiological evidence linking MASLD and HFpEF**.

Study Author	Study design	Study population	MASLD definition	Key findings
Fudim et al. [33]	Retrospective cohort	MASLD (n = 27,919) and non-MASLD (n = 842,616)	ICD-coded MASLD	Patients with MASLD were at an increased risk of incident HF (aHR 1.23, 95% CI 1.18–1.29), with a higher risk of developing HFpEF (aHR 1.24, 95% CI 1.14–1.34) compared with HFrEF (aHR 1.09, 95% CI 0.98–1.20).
Chang et al. [34]	Retrospective cohort	MASLD (n = 18,977) and non-MASLD (n = 7699)	ICD-coded MASLD	Patients with MASLD exhibited an increased risk of incident HF (sHR 2.59, 95% CI 1.84–3.64), with a specific independent association with HFpEF (sHR 1.91, 95% CI 1.27–2.86).
Kusner et al. [35]	Population-based prospective cohort	2932 MESA study participants	DLF defined by CT-based hepatic attenuation	Highest DLF quartile linked to adverse metabolic phenotype, cardiac remodeling, and elevated HFpEF risk.
Minhas et al. [36]	Retrospective cohort	HFpEF patients with MASLD (n = 32,875) and HFpEF patients without MASLD (1,328,865)	ICD-coded MASLD	HFpEF patients with MASLD had higher in-hospital mortality (aOR 1.65, 95% CI 1.43–1.90, *p* < 0.001), increased rates of adverse clinical events, longer hospital stay, and greater healthcare costs.
Petta et al. [37]	Prospective cohort	147 MASLD participants	Liver biopsy- proven MASLD	Patients with F3–F4 fibrosis, compared with those with F0–F2 fibrosis, exhibited lower ejection fraction (*p* = 0.004), reduced lateral TDI e′ (*p* = 0.009), decreased E/A ratio (*p* = 0.04), and a trend toward higher E/e′ (*p* = 0.07).
Miller et al. [38]	Retrospective cohort	HFpEF patients with MASLD (n = 49) and HFpEF patients without MASLD (n = 133)	Liver biopsy, abdominal imaging or ICD-coding MASLD	Patients with advanced fibrosis or cirrhosis, compared with those without, exhibited larger left atrial diameter (47 vs 43, *p *= 0.001) and a higher prevalence of ≥ grade 2 diastolic dysfunction (21vs 17, *p *= 0.04).
Chung et al. [39]	Retrospective cohort	3300 MASLD participants	Ultrasonography-defined MASLD	In the non-obese population, MASLD was associated with an incrementally increased risk of diastolic dysfunction according to fibrosis stage (aOR 1.40, 95% CI 1.06–1.84) for MASLD without advanced fibrosis and 1.44 (0.95–2.17) for MASLD with advanced fibrosis vs. no MASLD; *p* = 0.022.

MASLD, metabolic dysfunction–associated steatotic liver disease; ICD, international classification of diseases; HF, heart failure; aHR, adjusted hazard ratio; CI, confidence interval; HFpEF, heart failure with preserved ejection fraction; sHR, subdistribution hazard ratio; MESA, Multi-Ethnic Study of Atherosclerosis; DLF, disproportionate liver fat; CT, computed tomography; aOR, adjusted odds ratio.

## 4. Pathophysiological Links Between MASLD and HFpEF

The strong epidemiological association between MASLD and HFpEF necessitates an in-depth exploration of their pathophysiological links. A series of interconnected processes—including chronic low-grade inflammation, insulin resistance, lipotoxic stress, endothelial dysfunction, and gut-derived signaling—act synergistically, ultimately leading to adverse cardiac remodeling and impaired diastolic function. A thorough understanding of these mechanisms is critical, not only to elucidate how MASLD drives the onset and progression of HFpEF but also to provide a theoretical foundation for identifying potential therapeutic targets. The following sections delineate the major mechanistic axes linking MASLD to HFpEF, with an emphasis on how their interactions collectively shape disease initiation and phenotypic heterogeneity.

### 4.1 Low-grade Systemic Inflammation and Immune Dysregulation

Low-grade systemic inflammation is increasingly recognized as a central mediator linking MASLD to HFpEF. In patients with MASLD, particularly once it progresses to metabolic dysfunction–associated steatohepatitis (MASH), excessive hepatic lipid accumulation triggers oxidative stress, hepatocyte injury and death, the recruitment of inflammatory cells, and the increased secretion of pro-inflammatory mediators [40]. Hepatic immune cell populations—including Kupffer cells, dendritic cells, and macrophages—undergo phenotypic shifts from an immune-tolerant state to an immunogenic state during disease progression [41]. This pro-inflammatory reprogramming and activation of immune responses increase cytokine production and release, promote the infiltration of circulating immune cells into the liver, and amplify local inflammatory signaling [42]. Large amounts of inflammatory mediators subsequently enter the systemic circulation via the hepatic sinusoids [43]. Increased circulating levels of tumor necrosis factor-α (TNF-α), C-reactive protein (CRP), interleukin (IL)-6, and other pro-inflammatory factors exacerbate systemic inflammation, induce endothelial activation, alter leukocyte trafficking, and establish a globally pro-inflammatory milieu, thereby predisposing patients to cardiovascular structural and functional abnormalities [44].

Beyond systemic inflammation, epicardial adipose tissue (EAT) plays a critical role in the association between MASLD and HFpEF. As a metabolically active visceral fat depot in direct anatomical contact with the myocardium, EAT exerts local paracrine and vasocrine effects by secreting pro-inflammatory cytokines, adipokines, and profibrotic factors [45]. Multiple clinical studies have demonstrated that, compared with healthy individuals, patients with MASLD have increased EAT volume, and the degree of EAT expansion is correlated with the severity of hepatic steatosis and fibrosis [46,47,48]. EAT expansion is typically accompanied by the upregulation of pro-inflammatory cytokine expression and the infiltration of immune cells, including macrophages, dendritic cells, and T lymphocytes. Accordingly, EAT has emerged as a major source of pro-inflammatory and profibrotic mediators, such as IL-6, monocyte chemoattractant protein-1, TNF-α, and IL-1β [49]. These mediators promote ventricular fibrosis and coronary microvascular dysfunction, thereby contributing to the development and progression of HFpEF [45,50,51]. Several studies have reported that dysfunctional EAT, owing to its close anatomical proximity to the myocardium, can induce myocardial remodeling characterized by impaired diastolic function, ultimately leading to HFpEF [52]. Conversely, the removal of EAT has been shown to improve vascular architecture and restore diastolic function [53,54].

Collectively, chronic low-grade inflammation drives endothelial dysfunction, perturbs cardiomyocyte signaling, and promotes extracellular matrix remodeling through pro-inflammatory and pro-fibrotic pathways. Notably, this inflammatory milieu interacts with systemic insulin resistance, oxidative stress, and other metabolic derangements, resulting in a metabolically driven cardiac phenotype that is characteristic of HFpEF and is distinct from traditional ischemic or pressure overload–induced cardiomyopathies.

### 4.2 Systemic IR and Loss of Metabolic Flexibility

IR represents a central pathophysiological node linking MASLD and HFpEF. Beyond its canonical role in glucose homeostasis, systemic IR—often manifesting as hyperinsulinemia—has been identified as an independent risk factor for the development and progression of HFpEF, particularly among patients with cardiometabolic phenotypes characterized by obesity, T2DM, and MASLD [55]. In patients with MASLD, impaired insulin signaling promotes increased hepatic de novo lipogenesis and reduces lipid export, leading to excessive intracellular lipid accumulation within hepatocytes [56]. Concomitantly, IR disrupts triglyceride metabolism, resulting in elevated levels of very-low-density lipoprotein (VLDL) and small dense low-density lipoprotein (sdLDL), both of which are independent contributors to coronary artery disease and HF [57,58]. In response to this dysmetabolic state, the liver secretes a spectrum of prothrombotic and pro-inflammatory mediators, including fibrinogen, transforming growth factor-β (TGF-β), plasminogen activator inhibitor-1 (PAI-1), IL-6, TNF-α, and reactive oxygen species, further amplifying systemic metabolic dysfunction [59,60]. This process is exacerbated by enhanced adipose tissue lipolysis in the context of IR, which increases circulating free fatty acid (FFA) levels [59,61]. Excess FFAs induce cardiac lipotoxicity, impair myocardial energy metabolism, and promote diastolic dysfunction [62].

In the healthy myocardium, metabolic flexibility—the capacity to dynamically switch among fatty acids, glucose, lactate, ketone bodies, and amino acids as energy substrates—enables efficient adenosine triphosphate (ATP) generation across varying workload and nutrient availability conditions [63]. However, this finely tuned regulatory balance is disrupted under conditions of IR. Impaired insulin signaling attenuates glucose oxidation, driving the myocardium toward an increased reliance on glycolysis and ketone body oxidation for energy production [64,65,66]. Although this metabolic shift may initially be adaptive, it ultimately becomes maladaptive, as these alternative pathways require higher oxygen consumption and yield less ATP per molecule of substrate than glucose oxidation does [64,65,66]. The resulting metabolic inefficiency exacerbates myocardial energy depletion and leads to oxidative stress, mitochondrial dysfunction, and cardiomyocyte apoptosis [66,67]. Moreover, IR impairs the phosphoinositide 3-kinase/protein kinase B (PI3K/PKB) signaling pathway in cardiomyocytes, reduces nitric oxide (NO) bioavailability, and accelerates pathological cardiac remodeling [68,69,70]. Thus, loss of metabolic flexibility refers not only to the energetic substrate consumed for increased myocardial workload in HFpEF patients but also to its interaction with inflammatory and endothelial dysfunction pathways to promote myocardial fibrosis and increased ventricular stiffness.

Viewing IR and metabolic inflexibility as active drivers—rather than passive epiphenomena—of HFpEF underscores an urgent therapeutic imperative: in patients with MASLD-associated HFpEF, interventions should be used to restore substrate adaptability and enhance myocardial energetic resilience.

### 4.3 Hepatogenic Lipotoxicity and Myocardial Remodeling

MASLD is increasingly recognized as a major source of systemic lipotoxic stress rather than a condition confined to hepatic lipid accumulation. Under conditions of chronic energy surplus and IR, the steatotic liver becomes a primary exporter of bioactive lipids, including FFAs, diacylglycerols, ceramides, and triglyceride-rich lipoprotein remnants [71]. These lipid intermediates act as signaling molecules that disrupt cellular homeostasis in distant organs, establishing a liver-derived lipotoxic state that extends beyond the liver itself [72].

At the hepatic level, excessive lipid influx combined with impaired β-oxidation promotes the intracellular accumulation of toxic lipid intermediates, triggering endoplasmic reticulum stress, mitochondrial dysfunction, and the activation of stress-responsive pathways such as c-Jun N-terminal kinase (JNK), nuclear factor kappa-B (NF-κB), and the nucleotide-binding domain, leucine-rich-containing family, pyrin domain-containing 3 (NLRP3) inflammasome [73,74,75,76,77]. Although triglyceride sequestration within lipid droplets may initially exert a protective effect, progressive MASLD is characterized by an imbalance in which the generation of lipotoxic species exceeds the capacity for neutral lipid storage [71]. Consequently, the liver releases increased amounts of FFAs and apolipoprotein-containing lipoproteins into the systemic circulation, exacerbating whole-body lipid oversupply and chronic exposure of the myocardium to lipotoxic injury.

In the heart, excessive uptake of circulating FFAs and lipid intermediates leads to myocardial steatosis, a phenomenon commonly observed in individuals with obesity, T2DM, and HFpEF [62,78,79]. Given the high energy demands and limited lipid storage capacity of cardiomyocytes, these cells are particularly vulnerable to lipotoxic stress. The intracellular accumulation of ceramides and diacylglycerols in cardiomyocytes impairs insulin signaling, disrupts mitochondrial oxidative phosphorylation, and increases reactive oxygen species production [80,81]. These metabolic perturbations reduce ATP availability and disproportionately compromise energy-dependent diastolic relaxation, which is a hallmark of HFpEF. In addition, ceramide-mediated mitochondrial dysfunction and the activation of pro-apoptotic pathways result in cardiomyocyte loss and replacement fibrosis, further increasing myocardial stiffness [82].

Moreover, liver-derived lipotoxicity acts synergistically with vascular dysfunction. Lipid intermediates decrease endothelial nitric oxide bioavailability and promote endothelial inflammation, leading to coronary microvascular dysfunction and capillary rarefaction [83,84,85]. Reduced microvascular reserve limits oxygen and substrate delivery to cardiomyocytes, intensifies metabolic stress, and reinforces maladaptive cardiac remodeling.

In summary, MASLD-driven lipotoxicity constitutes a core mechanistic axis that links hepatic metabolic dysfunction to myocardial remodeling in HFpEF patients. Lipotoxic signaling does not operate in isolation but instead integrates with chronic low-grade inflammation, IR, and endothelial dysfunction to generate a distinct cardiometabolic remodeling phenotype characterized by increased myocardial stiffness, impaired diastolic function, and preserved systolic performance.

### 4.4 Endothelial Dysfunction and Microvascular Rarefaction

MASLD establishes a systemic environment characterized by chronic low-grade inflammation, IR, and lipotoxic stress, each of which has profound detrimental effects on the vascular endothelium. Endothelial dysfunction has emerged as a critical interface through which systemic metabolic disturbances translate into adverse alterations in myocardial structure and function [86].

NO, a key endothelium-derived vasodilator, is essential for maintaining endothelial health because it promotes vasodilation, inhibits platelet aggregation, and suppresses the proliferation of smooth muscle cells. In MASLD patients, IR impairs the PI3K/PKB signaling pathway, resulting in reduced endothelial nitric oxide synthase (eNOS) activation and decreased NO bioavailability [68]. Reduced NO bioavailability exerts a cascade of downstream effects on the myocardial function. Specifically, NO deficiency limits the activation of soluble guanylate cyclase (sGC) in cardiomyocytes, leading to decreased cyclic guanosine monophosphate (cGMP) levels and suppressed protein kinase G (PKG) activity [87]. Defects in this signaling axis have been confirmed in myocardial tissues from HFpEF patients, and the cGMP content and PKG activity in these tissues are significantly lower [88]. Extensive evidence has indicated that PKG plays a pivotal role in inhibiting cardiomyocyte hypertrophy. For example, in neonatal rat cardiomyocyte models, NO or cGMP analogs attenuate norepinephrine-induced hypertrophic responses [89]. Clinically, compared with that in patients with HFrEF, myocardial PKG activity in HFpEF patients is lower, and the degree of reduction is closely correlated with increased cardiomyocyte diameter, linking diminished PKG activity to hypertrophy and concentric remodeling [88]. In addition to structural remodeling, disruption of the NO–cGMP–PKG pathway directly impairs diastolic function. This signaling defect increases diastolic cytosolic Ca^2+^ levels via mechanisms including increased protein phosphatase 2A activity and reduced sarcoplasmic reticulum Ca^2+^ reuptake, thereby compromising myocardial relaxation [90,91]. Additionally, the pathway regulates cardiomyocyte stiffness through the phosphorylation of sarcomeric proteins, notably titin. PKG-dependent phosphorylation of the titin N2B segment increases cardiomyocyte compliance, whereas PKG expression deficiency results in hypophosphorylated titin and elevated resting tension [88,92,93]. Cardiomyocytes isolated from HFpEF patients exhibit significantly increased resting tension, which can be experimentally reversed by restoring PKG activity [88,94,95]. These findings provide a direct mechanistic link between endothelial NO deficiency and increased intrinsic myocardial stiffness.

Concomitantly, MASLD-associated systemic inflammation exacerbates endothelial dysfunction by upregulating the expression of adhesion molecules such as vascular cell adhesion molecule-1 (VCAM-1), intercellular adhesion molecule-1 (ICAM-1), and E-selectin, promoting leukocyte adhesion and transmigration into the vascular wall [96,97,98]. Chronic endothelial activation intensifies microvascular inflammation, which has been proposed as a unifying mechanism underlying HFpEF pathogenesis [86]. Persistent microvascular inflammation and oxidative stress ultimately lead to structural remodeling characterized by progressive capillary rarefaction [96]. Histopathological and functional studies in HFpEF patients have demonstrated that reduced coronary microvascular density is closely correlated with the severity of diastolic dysfunction and impaired exercise capacity [99]. Microvascular rarefaction limits oxygen and nutrient delivery to cardiomyocytes, exacerbating metabolic stress and mitochondrial dysfunction [99]. In the context of MASLD, hepatic IR and atherogenic dyslipidemia amplify these processes.

Overall, endothelial dysfunction and microvascular rarefaction constitute a vasculature-centered mechanistic pathway through which MASLD-driven metabolic and inflammatory disturbances are translated into myocardial structural remodeling and diastolic dysfunction. This conceptual framework reinforces the notion that HFpEF is a manifestation of systemic cardiometabolic dysregulation.

### 4.5 Hepatokines

Beyond its metabolic and inflammatory roles, the liver functions as an endocrine organ that secretes a spectrum of hepatokines, which play critical roles in mediating inter-organ communication along the liver–heart axis. In the setting of MASLD, dysregulated hepatokine secretion contributes to systemic metabolic disturbance, low-grade inflammation, and direct myocardial injury, thereby promoting the development of HFpEF [100].

Fibroblast growth factor 21 (FGF21) has emerged as a pivotal metabolic regulator mediating liver–heart crosstalk [101,102]. In MASLD, circulating FGF21 levels are consistently elevated and positively correlate with disease severity and progression [103]. Preclinical studies indicate that FGF21 exerts cardioprotective effects through multiple mechanisms, including enhancement of antioxidant defenses, promotion of fatty acid oxidation, and preservation of mitochondrial function [100]. However, persistent elevation of FGF21 under chronic metabolic stress may have paradoxical implications. Sustained high FGF21 levels have been implicated in maladaptive cardiac remodeling [104]. This apparent discrepancy may be explained by the concept of “FGF21 resistance”, characterized by impaired receptor complex function and blunted downstream signaling [105]. In this context, elevated FGF21 levels likely reflect a compensatory response to metabolic stress and endocrine dysregulation, rather than effective cardioprotection.

Liver-derived mediators, including coagulation factor XI and serum amyloid A (SAA1 and SAA4), have recently been identified as novel links between metabolic liver disease and cardiac dysfunction. Preclinical studies indicate that factor XI exerts cardioprotective effects in mice by activating the bone morphogenetic protein –SMAD1/SMAD5 signaling pathway in cardiomyocytes, thereby attenuating inflammation and fibrosis and inhibiting the development of HFpEF [106]. Consistently, clinical observations show reduced plasma levels of factor XI in patients with HFpEF, suggesting its involvement in the pathophysiology of the disease [106]. Similarly, SAA1 and SAA4, both acute-phase proteins, display a distinctive overlap in plasma proteomic profiles of patients with MASLD and HFpEF and are associated with upregulated transcription of genes involved in extracellular matrix remodeling in the heart [107]. In addition, fetuin-A, an endogenous inhibitor of insulin receptor tyrosine kinase, facilitates the binding of circulating free fatty acids to Toll-like receptor 4 (TLR4), thereby promoting pro-inflammatory signaling [108]. Elevated circulating fetuin-A levels are independently associated with metabolic syndrome and adverse cardiometabolic outcomes [109]; however, its role in HFpEF remains to be fully elucidated. Further studies are warranted to clarify the mechanistic contributions of these relatively underexplored hepatokines in mediating the interplay between MASLD and HFpEF.

Collectively, these hepatokines form a complex endocrine network through which hepatic metabolic dysfunction is translated into cardiac structural and functional abnormalities. This hepatokine-mediated signaling axis provides a mechanistic link between MASLD and HFpEF and may represent a promising source of biomarkers and therapeutic targets.

### 4.6 Gut–Liver–Heart Crosstalk

Recent studies have highlighted the complex interplay between the gut microbiota, MASLD, and cardiovascular risk, suggesting that the gut microbiome plays a pivotal role in the development and progression of these conditions. In MASLD patients, the gut microbial composition is significantly altered from that of healthy individuals, accompanied by impaired intestinal barrier integrity, which facilitates the translocation of microbe-derived metabolites and inflammatory mediators into the portal circulation. These factors directly influence hepatic inflammatory responses and systemic cardiometabolic homeostasis.

Gut dysbiosis in MASLD patients is characterized by a decrease in the abundance of beneficial bacteria, such as *Akkermansia* and *Prevotella*, and an enrichment of potentially pathogenic species [110,111]. This imbalance increases intestinal permeability, allowing bacterial products such as lipopolysaccharide (LPS) to enter the portal vein and activate hepatic Kupffer cells and stellate cells through Toll-like receptor signaling. This process not only exacerbates hepatic inflammation and fibrosis but also promotes the release of pro-inflammatory cytokines and liver-derived factors into the systemic circulation, thereby contributing to myocardial fibrosis and ventricular stiffening [110,112].

Beyond classical inflammatory mediators, gut microbiota–derived metabolites have emerged as key mechanistic intermediates linking MASLD to HFpEF [113,114]. Among these, trimethylamine N-oxide (TMAO), short-chain fatty acids (SCFAs), and bile acids (BAs) are the most extensively studied. TMAO, produced via hepatic oxidation of gut-derived trimethylamine, is associated with endothelial dysfunction, vascular inflammation, and adverse cardiovascular outcomes in HFpEF patients [115]. Mechanistically, TMAO promotes endothelial dysfunction by impairing nitric oxide bioavailability, enhances vascular inflammation through activation of NF-κB signaling, and drives myocardial fibrosis via transforming growth factor-β (TGF-β)/Smad signaling pathways, ultimately contributing to increased myocardial stiffness and impaired relaxation—hallmark features of HFpEF [116,117,118]. In contrast, SCFAs—including acetate, propionate, and butyrate—generally exert protective cardiometabolic effects under physiological conditions. SCFAs enhance endothelial function, improve insulin sensitivity, and attenuate systemic inflammation through activation of G protein-coupled receptors (GPR41 and GPR43) [119,120]. However, in MASLD-associated dysbiosis, reduced SCFA production or impaired signaling may weaken these protective mechanisms, thereby indirectly promoting endothelial dysfunction, microvascular inflammation, and ventricular stiffening [121]. BAs, synthesized in the liver and converted to secondary BAs by the gut microbiota, participate in BA metabolism [122]. BAs further contribute to gut–liver–heart signaling via activation of nuclear and membrane receptors, particularly farnesoid X receptor (FXR) and Takeda G protein-coupled receptor 5 (TGR5) [123,124,125]. Dysregulated bile acid signaling in MASLD has been linked to altered lipid metabolism, oxidative stress, and inflammatory activation [123,124,125]. Emerging evidence suggests that impaired FXR/TGR5 signaling may contribute to cardiac remodeling and diastolic dysfunction by modulating myocardial energy metabolism and inflammatory responses [126,127].

Collectively, the “gut–liver–heart axis” provides a unifying framework for understanding MASLD-related HFpEF, linking gut microbial dysbiosis, hepatic inflammation, endothelial dysfunction, and myocardial remodeling. Within this axis, the liver functions not only as a central metabolic organ but also as an amplifier of harmful gut-derived signals, whereas the heart serves as a critical target of chronic metabolic and inflammatory stress.

### 4.7 Integrative Role of T2DM in MASLD–HFpEF Pathophysiology

Collectively, the aforementioned mechanisms linking MASLD to HFpEF do not operate in isolation but converge within a shared cardiometabolic milieu, in which T2DM functions as a central integrator and amplifier of multi-organ dysfunction. Rather than representing a parallel comorbidity, T2DM provides a unifying pathophysiological context that intensifies the interconnected processes of metabolic dysregulation, chronic inflammation, lipotoxic stress, endothelial dysfunction, and gut–liver–heart crosstalk [128].

In the diabetic state, profound insulin resistance and persistent hyperglycemia exacerbate hepatic lipid accumulation, promote glucotoxicity, and impair systemic substrate utilization, thereby reinforcing metabolic inflexibility across both hepatic and myocardial tissues [129]. Concurrently, T2DM is associated with sustained activation of pro-inflammatory pathways and increased circulating cytokines, amplifying the low-grade inflammatory milieu that underlies both MASLD progression and HFpEF development [128,130]. Endothelial dysfunction is likewise aggravated in T2DM, as impaired nitric oxide bioavailability, oxidative stress, and diabetes-associated dyslipidemia collectively accelerate coronary microvascular dysfunction and capillary rarefaction—key determinants of myocardial stiffness and diastolic impairment [68,69,70,86]. T2DM also amplifies inter-organ crosstalk within the gut–liver–adipose–heart axis. In the diabetic state, adipose tissue dysfunction and increased lipolysis lead to excessive free fatty acid flux to the liver and heart, exacerbating hepatogenic lipotoxicity and myocardial lipid accumulation [59,61]. Meanwhile, diabetes-associated gut microbiota alterations and increased intestinal permeability enhance the translocation of microbial metabolites such as trimethylamine N-oxide and endotoxins, further promoting systemic inflammation, endothelial dysfunction, and myocardial fibrosis [110,112,131]. These multi-organ interactions establish a feed-forward cycle in which hepatic, metabolic, and cardiovascular abnormalities mutually reinforce one another.

This integrative framework provides a mechanistic basis for the disproportionately high risk of HFpEF and adverse cardiovascular outcomes observed in patients with MASLD and T2DM, and helps explain why disease severity—particularly fibrosis burden—rather than steatosis alone, more strongly predicts clinical outcomes. Recognizing T2DM as a central pathophysiological hub underscores the importance of targeting upstream metabolic dysfunction and supports the development of phenotype-oriented therapeutic strategies in MASLD-associated HFpEF.

**Fig. 1.**
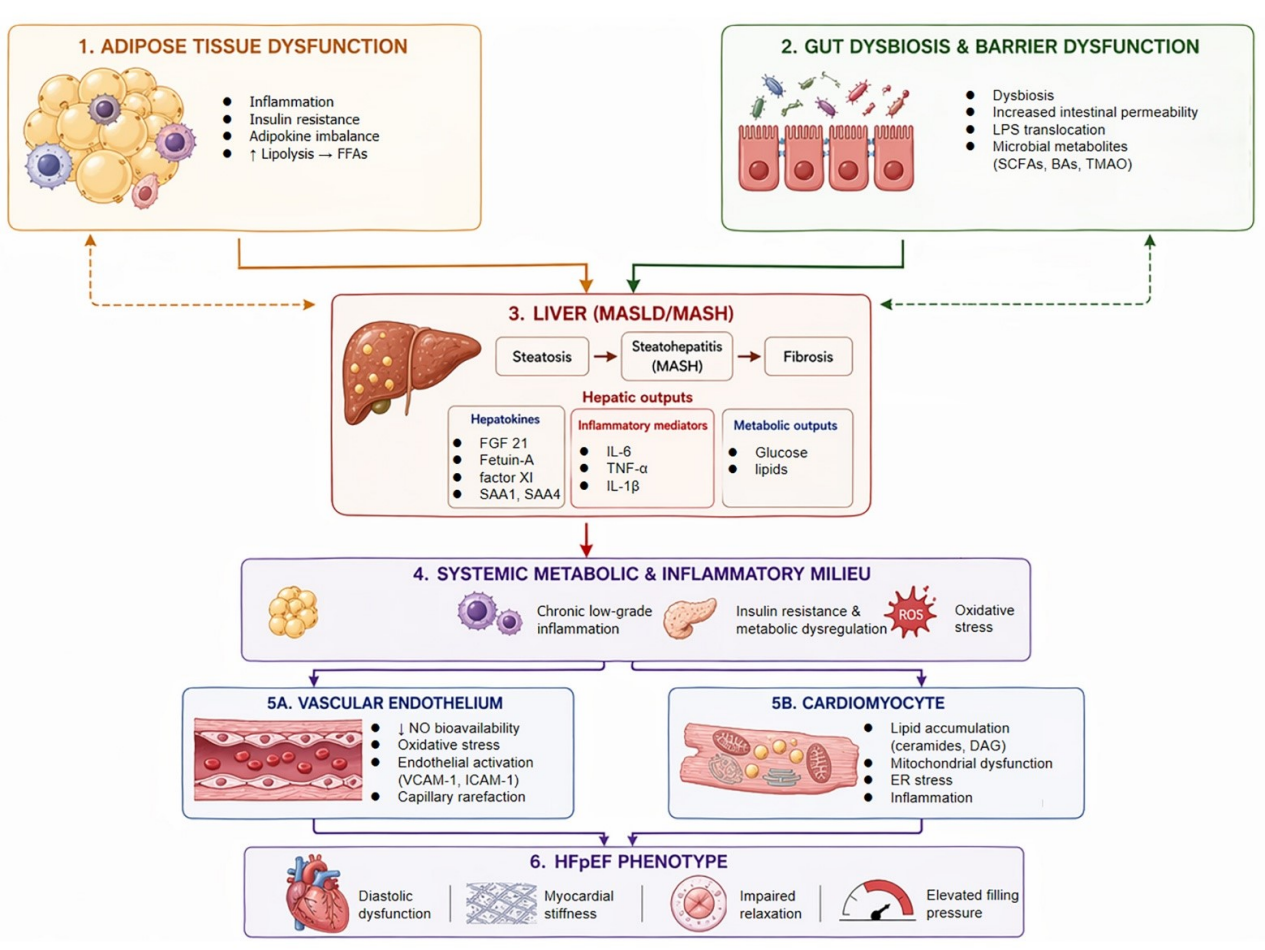
**Pathophysiology of MASLD and HFpEF**. Adipose tissue dysfunction and gut dysbiosis act upstream to promote MASLD development and progression. The diseased liver releases hepatokines, inflammatory cytokines, and metabolic products, generating a systemic milieu of lipotoxicity, inflammation, insulin resistance, and oxidative stress. These circulating mediators impair coronary microvascular endothelial function and induce cardiomyocyte injury, ultimately leading to the HFpEF phenotype.

Mechanistic links between MASLD and HFpEF are shown in Fig. 1.

## 5. Diagnosis and Risk Stratification in MASLD and HFpEF

Recognition of the substantial epidemiological overlap and shared mechanistic underpinnings between MASLD and HFpEF supports the implementation of a unified diagnostic pathway and integrated multi-organ assessment from the initial clinical encounter in patients with cardiometabolic disease. Diagnostic algorithms for MASLD and HFpEF have been incorporated into respective specialty guidelines [9,132,133]. However, current guidelines for either condition do not explicitly recommend reciprocal evaluation. Expanding pragmatic diagnostic pathways to incorporate multi-organ assessment is therefore critical for early identification, therapeutic decision-making, and prognostic stratification.

### 5.1 Imaging Modalities

Ultrasonography. Transthoracic echocardiography remains the cornerstone for the diagnosis of HFpEF [9]. Similarly, B-mode hepatic ultrasonography is the first-line modality for detecting hepatic steatosis [134]. Given the increased risk of HFpEF among patients with MASLD—and vice versa—concomitant cardiac and hepatic ultrasound assessment may facilitate a more systematic diagnostic approach. When HFpEF is strongly suspected but resting echocardiographic findings are inconclusive, diastolic stress imaging is recommended as a second-line evaluation [135].

Magnetic resonance imaging (MRI). Multiparametric cardiac magnetic resonance (CMR) enables precise characterization of cardiac structure and function in HFpEF [136,137,138,139]. Magnetic resonance imaging is likewise highly informative for hepatic assessment. Liver MRI represents the most comprehensive imaging modality for screening and phenotyping MASLD [132,140]. In particular, proton density fat fraction (PDFF) MRI is the noninvasive reference standard for quantitative assessment of hepatic steatosis in both clinical and research settings [141].

Overall, steatosis and fibrosis are hallmark features of both HFpEF and MASLD. MRI allows highly accurate quantification of lipid content and fibrosis in both the heart and liver; when dual pathology is clinically suspected, MRI may serve as a definitive diagnostic modality.

### 5.2 Biomarkers and Diagnostic Scores

Although ultrasonography and MRI are essential for the diagnosis of HFpEF and MASLD, they are not well-suited for population-level screening. Accordingly, diagnostic scores integrating clinical history, phenotypic features, and laboratory parameters offer greater scalability.

HFpEF. In the absence of overt congestion, elevated plasma natriuretic peptide levels represent the most widely used biomarker for HFpEF [133]. Despite guideline recommendations, established thresholds may fail to identify a subset of patients, particularly those with obesity, in whom natriuretic peptide levels are often normal or only mildly elevated despite increased cardiac filling pressures [142,143,144]. Clinical scoring systems such as H_2_FPEF [145] and HFA-PEFF [135] have been developed to support the diagnosis of HFpEF. A simplified algorithm incorporating only body mass index, age, and the presence of atrial fibrillation (HFpEF-ABA score) has also been proposed to identify individuals at high risk [146]. While these scores are not diagnostic gold standards, they may facilitate risk stratification of patients with MASLD within an integrated, multi-organ assessment framework.

MASLD. Multiple clinical scores have been developed to detect and stage hepatic steatosis [147,148]. In addition, screening for liver fibrosis is essential for identifying patients at high risk of liver-related outcomes and is therefore recommended in MASLD [132]. The most widely endorsed fibrosis scores in international guidelines include the fibrosis-4 index (FIB-4) [149], the aspartate aminotransferase-to-platelet ratio index (APRI) [150], and the NAFLD fibrosis score (NFS) [151]. These tools rely on routinely available clinical parameters, rendering them suitable for both population-based screening and routine clinical practice.

### 5.3 Integrated Diagnostic Pathway

**Fig. 2.**
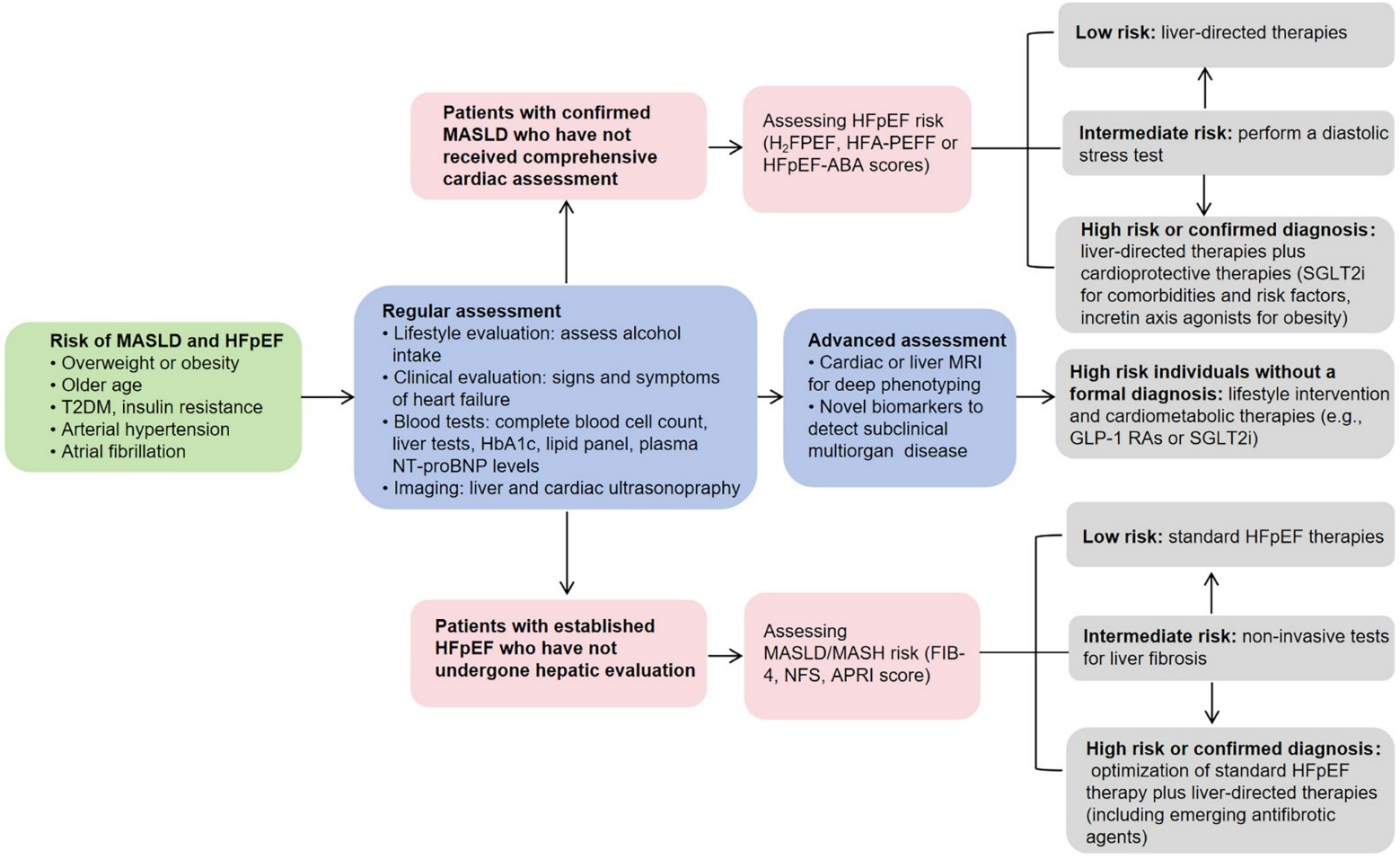
**Integrated diagnostic and management algorithm for MASLD and HFpEF**. An integrated diagnostic approach for HFpEF and MASLD provides a structured framework for guiding therapeutic decision-making across different clinical scenarios. This strategy is particularly applicable to three patient groups: (i) individuals at risk of either condition without a formal diagnosis, (ii) patients with established HFpEF who have not undergone hepatic evaluation, and (iii) patients with confirmed MASLD who have not received comprehensive cardiac assessment. For at-risk individuals—such as those with obesity, advanced age, T2DM, hypertension, or atrial fibrillation—initial multidimensional evaluation is recommended. This includes clinical assessment, laboratory testing (liver function, glycemic and lipid profiles, and natriuretic peptides), and imaging studies (hepatic and cardiac ultrasonography). For patients with an established diagnosis of a single condition, targeted cross-system risk assessment should be performed. In patients with HFpEF, noninvasive fibrosis scores—such as FIB-4, NFS, or APRI—are recommended to evaluate MASLD and hepatic fibrosis risk. Conversely, in patients with MASLD, validated scoring systems including H_2_FPEF, HFA-PEFF, or HFpEF-ABA should be applied to stratify the risk of HFpEF. Based on risk stratification, individualized therapeutic strategies can then be implemented to optimize clinical outcomes. In addition, advanced imaging modalities, such as MRI, and emerging biomarker panels may provide further insights into subclinical multi-organ involvement and enhance prognostic accuracy.

An integrated diagnostic approach for HFpEF and MASLD may provide a more precise framework to guide therapeutic prioritization across these interrelated conditions (Fig. 2). This approach is particularly applicable in three clinical scenarios: (i) individuals at risk of either condition without a formal diagnosis, (ii) patients with established HFpEF who have not [35] undergone hepatic evaluation, and (iii) patients with confirmed MASLD who have not received comprehensive cardiac assessment.

## 6. Therapeutic Approaches for MASLD and HFpEF

Recognition of MASLD as a systemic driver of HFpEF has important therapeutic implications, shifting management beyond treatment of isolated cardiac targets toward modulation of the interconnected metabolic, inflammatory, and vascular networks linking the liver, adipose tissue, and myocardium. Accordingly, interventions that improve hepatic steatosis, insulin sensitivity, and systemic metabolic homeostasis may provide pleiotropic benefits for both hepatic and cardiovascular outcomes. Notably, T2DM, which is highly prevalent in both MASLD and HFpEF populations, represents a key determinant of therapeutic response and clinical prioritization. Accordingly, therapeutic interventions targeting shared cardiometabolic pathways may yield particularly pronounced benefits in patients with coexisting MASLD, HFpEF, and T2DM. In the following section, we review the emerging therapeutic approaches for MASLD and HFpEF, integrating the available clinical evidence with the underlying pathophysiological mechanisms.

### 6.1 Lifestyle Interventions

Weight reduction is critical for improving outcomes in patients with MASLD. Evidence indicates that a body weight loss of ≥5% can improve hepatic steatosis, whereas a reduction of ≥7% can alleviate MASH in most patients [152]. Notably, weight loss ≥10% may even lead to the regression of hepatic fibrosis [152]. These hepatic benefits are paralleled by cardiovascular improvements; large cohort studies have demonstrated that weight loss is independently associated with a reduced risk of heart failure [153,154]. Consequently, lifestyle interventions—including dietary modification and increased physical activity—not only represent a cornerstone of MASLD management but also play a pivotal role in the prevention and treatment of cardiovascular disease.

#### 6.1.1 Diet

Lifestyle interventions aimed at weight reduction remain the cornerstone of MASLD management. A 12-week randomized controlled trial (RCT) including 70 MASLD patients demonstrated that those undergoing Dietary Approaches to Stop Hypertension (DASH) diet intervention exhibited significant improvements in alanine aminotransferase (ALT) and aspartate aminotransferase (AST) levels, hepatic steatosis, and fibrosis compared with the control group [155]. A meta-analysis further indicated that low-carbohydrate diets significantly reduce hepatic fat content and improve liver enzyme levels in MASLD patients, with effects comparable to those of low-fat diets [156]. Another meta-analysis confirmed that calorie restriction interventions significantly improve ALT levels, hepatic steatosis, and liver stiffness, whereas adherence to a Mediterranean diet (MedDiet) reduces ALT levels, the fatty liver index, and liver stiffness [157]. In addition, intermittent fasting—including alternate-day fasting and other forms of periodic caloric restriction—has garnered increasing attention. A meta-analysis of 10 studies of adult MASLD patients revealed that intermittent fasting interventions significantly improved serum ALT and AST levels, hepatic steatosis, and liver stiffness, as measured by controlled attenuation parameter-based transient elastography [158].

Different dietary patterns also have important implications for patients with HFpEF. In a study of 13 hypertensive patients with HFpEF, DASH diet intervention significantly improved left ventricular diastolic function, arterial elasticity, and ventriculo-arterial coupling [159]. Another study of obese patients with T2DM reported that a low-energy meal replacement diet led to marked improvements in left ventricular mass/volume and aortic stiffness compared with those in controls [160]; however, dedicated dietary intervention studies in HFpEF patients remain limited. The MedDiet is considered to be cardioprotective [161]. The MEDIT-AHF study revealed that among patients hospitalized for acute heart failure, stricter adherence to the MedDiet was associated with significantly lower HF rehospitalization rates, although it did not reduce long-term mortality [162]. Similarly, a Greek HF cohort study (with 38% HFpEF patients) demonstrated that the MedDiet improved outcomes in patients with HFpEF [163].

#### 6.1.2 Exercise

Exercise training should be implemented according to current guideline recommendations for at least 150 min per week of moderate-intensity physical activity. Meta-analyses have shown that in MASLD patients, regular exercise alone—without dietary modification—significantly reduces intrahepatic lipid content and lowers ALT and AST levels, with individuals with higher BMIs potentially receiving greater benefits [164]. Another meta-analysis of 17 studies demonstrated that exercise training significantly reduces hepatic triglyceride levels while improving hepatic insulin sensitivity [165]. In patients with HFpEF, exercise improves diastolic function, enhances cardiorespiratory fitness and exercise capacity, and increases quality of life [166,167,168]. Some studies have also reported that exercise interventions may reduce hospitalization rates or cardiac events in HFpEF patients [169,170]. The structure and supervision of an exercise program significantly influence its outcomes; compared with self-directed activity, supervised exercise yields superior results related to weight reduction and heart failure risk mitigation [171,172].

Comprehensive lifestyle programs have demonstrated greater efficacy than single interventions. A recent meta-analysis indicated that combined dietary and exercise interventions in MASLD patients outperform either intervention alone in improving liver function markers and insulin sensitivity [173]. Furthermore, the Look AHEAD trial investigated whether an intensive lifestyle intervention—targeting ≥10% weight loss through a structured diet and exercise combined with regular behavioral support—could reduce cardiovascular events compared with standard diabetes support and education [174]. Although the trial did not show an overall significant difference in the primary composite cardiovascular endpoint, participants who achieved substantial weight loss exhibited a lower HFpEF incidence, mediated by reductions in visceral adiposity [175] and hepatic triglyceride content [176].

In summary, intensive lifestyle interventions are critical strategies to prevent or delay the progression of MASLD and HFpEF. These interventions primarily target shared cardiometabolic pathways, including reducing systemic inflammation, improving insulin sensitivity, and decreasing visceral adiposity. However, the effective implementation and long-term maintenance of meaningful lifestyle changes remain challenging, highlighting the need for additional adjunctive therapeutic strategies.

### 6.2 Pharmacological Interventions

Over the past two decades, pharmacotherapy for T2DM has undergone a profound transformation. The advent of incretin-based therapies and sodium–glucose cotransporter 2 inhibitors (SGLT2i) has not only provided clinicians with safe and effective strategies for glycaemic control, but also enabled the treatment of underlying metabolic disturbances driven by obesity, insulin resistance, and MASLD. Importantly, these agents have demonstrated clinically meaningful benefits in patients with HFpEF. Accordingly, antidiabetic drugs with well-defined cardiometabolic effects have emerged as promising therapeutic candidates targeting the shared pathophysiological pathways linking MASLD and HFpEF. The following section will focus on the principal pharmacological classes that may confer dual benefits in hepatic steatosis and HFpEF, with an emphasis on their mechanistic underpinnings and emerging clinical evidence.

Clinical evidence on Glucagon-like Peptide-1 Receptor Agonists (GLP-1RAs) and SGLT2i in MASLD and HFpEF is summarized in Table 2 (Ref. [177,178,179,180,181,182,183,184,185,186]).

**Table 2.** **Clinical evidence for GLP-1RAs and SGLT2i in MASLD and HFpEF**.

**A. MASLD / MASH Clinical Trials**					
**Drug/Class**	**Trial**	**Medication**	**Population**	**Primary Endpoint**	**Key Findings**
GLP-1RAs	LEAN [177]	Liraglutide 1.8 mg sc once per day vs. placebo	52 Biopsy-proven MASH patients	MASH resolution	Histological MASH resolution in 39% of the liraglutide group vs 9% placebo
	SYNERGY [178]	Tirzepatide up to 15 mg sc once per week vs. placebo	157 Biopsy-proven MASH patients with fibrosis	MASH resolution without fibrosis worsening	MASH resolution without fibrosis worsening in 44–62% of tirzepatide-treated participants vs 10% on placebo
	ESSENCE interim analysis [179]	Semaglutide 2.4 mg sc once per week vs. placebo	800 Biopsy-proven MASH patients with fibrosis	MASH resolution without fibrosis worsening	MASH resolution without fibrosis progression in 62.9% of the semaglutide group vs 34.3% placebo
	A randomized phase 2a trial [180]	Retatrutide up to 12 mg sc once per week vs. placebo	98 MRI-PDFF-diagnosed MASLD patients	Change in liver fat	Significantly reduced hepatic fat: up to 82.4% with 12 mg dose
SGLT2i	A Meta-Analysis of RCT [184]	Sotagliflozin, dapagliflozin, empagliflozin et al.	573 imaging-diagnosed MASLD patients with T2DM	-	Significantly reduced ALT (WMD 5.36, 95% CI 8.86−1.85, *p* = 0.003), AST (WMD 2.56, 95% CI 3.83−1.29, *p* < 0.0001), and liver proton density fat fraction (WMD 2.20, 95% CI 3.67−0.74, *p* = 0.003)
**B. HFpEF Clinical Trials**					
**Drug/Class**	**Trial**	**Medication**	**Population**	**Primary Endpoint**	**Key Findings**
GLP-1RAs	STEP-HFpEF [181]	Semaglutide 2.4 mg sc once per week vs. placebo	529 obese-HFpEF patients	Change in KCCQ-CSS and body weight	(1) 7.8 points estimated difference in KCCQ from baseline to Week 52 (2) 10.7 estimated difference in body weight from baseline to Week 52
	STEP-HFpEF DM [182]	Semaglutide 2.4 mg sc once per week vs. placebo	616 obese-HFpEF patients with T2DM	Change in KCCQ- CSS and body weight	(1) 7.3 points estimated difference in KCCQ from baseline to Week 52 (2) 6.4 estimated difference in body weight from baseline to Week 52
	SUMMIT [183]	Tirzepatide up to 15 mg sc once per week vs. placebo	731 obese-HFpEF patients (with or without T2DM)	Cardiovascular death or HF worsening	The composite endpoint of HF worsening or cardiovascular death significantly improved from baseline to a median of 104 weeks (HR 0.62, 95% CI 0.41–0.95, *p* = 0.026)
SGLT2i	EMPEROR-Preserved [185]	Empagliflozin 10 mg po once per day vs. placebo	5988 HFpEF patients (with or without T2DM)	Cardiovascular death or HF hospitalization	The composite endpoint risk of cardiovascular death or HF hospitalization significantly reduced from baseline to a median of 26.2 months (HR 0.79, 95% CI 0.69–0.90, *p* < 0.001)
	DELIVER [186]	Dapagliflozin 10 mg po once per day vs. placebo	6263 HFpEF patients (with or without T2DM)	worsening HF or cardiovascular death	The composite endpoint of HF worsening or cardiovascular death significantly reduced from baseline to a median of 2.3 years (HR 0.82, 95% CI 0.73–0.92, *p* < 0.001)

GLP-1RAs, glucagon-like peptide-1 receptor agonists; SGLT2i, sodium–glucose cotransporter 2 inhibitors; MASLD, metabolic dysfunction–associated steatotic liver disease; HFpEF, heart failure with preserved ejection fraction; MASH, metabolic dysfunction–associated steatohepatitis; MRI-PDFF, Magnetic Resonance Imaging-Proton Density Fat Fraction; RCT, randomized controlled trial; T2DM, type 2 diabetes mellitus; KCCQ-CSS, Kansas City Cardiomyopathy Questionnaire clinical summary score; HF, heart failure.

#### 6.2.1 GLP-1RAs

6.2.1.1 Clinical Evidence Regarding GLP-1RAs in MASLD and HFpEF Patients

GLP-1RAs, dual GLP-1/gastric inhibitory polypeptide (GIP) receptor agonists, and triple GLP-1/GIP/glucagon receptor agonists have emerged as transformative therapies for the management of metabolic diseases, demonstrating pleiotropic benefits across multiple organ systems. Among GLP-1RAs, semaglutide has the most pronounced weight-reducing effect, with clinical trials reporting body weight reductions of up to 15% [187]. The dual GLP-1/GIP receptor agonist tirzepatide confers even greater weight loss: In the SURMOUNT-1 trial, approximately 57% of participants receiving the 15.0 mg weekly dose achieved ≥20% weight loss [188]. Furthermore, the novel triple agonist retatrutide demonstrated superior efficacy in a 48-week phase 2 trial, with maximal weight loss reaching 24.2% [189].

Beyond sustained weight reduction, GLP-1RAs have substantial therapeutic effects in patients with steatohepatitis. The LEAN trial (including individuals without T2DM) revealed that 39% of participants treated with liraglutide achieved histological resolution of MASH, compared with only 9% in the placebo group [177]. The SYNERGY trial further confirmed the benefits of tirzepatide in patients with MASH: after 52 weeks of treatment, 44–62% of participants achieved MASH resolution without worsening fibrosis in a clear dose-dependent manner [178]. Similarly, the ESSENCE interim analysis of semaglutide in MASH demonstrated that among 534 treated patients, 62.9% achieved MASH resolution without fibrosis progression, compared with 34.3% in the placebo group (n = 266) [179]. In addition, retatrutide treatment significantly reduced hepatic fat content, with a maximal reduction of 82.4% in the 12 mg dose group; reductions in liver fat were closely associated with improvements in insulin sensitivity and lipid metabolism [180]. Collectively, these findings establish GLP-1–based therapies as effective pharmacological options for patients with MASLD.

In addition to hepatic benefits, the cardiovascular advantages of GLP-1RAs have been consistently demonstrated across multiple trials. The SELECT trial, which included more than 4000 patients with HF, revealed that semaglutide treatment reduced the risk of a composite HF endpoint by 18%, accompanied by fewer absolute events of HF-related hospitalization or urgent HF-related visits [190]. Importantly, the STEP-HFpEF and STEP-HFpEF DM trials demonstrated that semaglutide significantly improved HF symptoms, physical limitations, and exercise capacity in patients with HFpEF [181,182]. Similarly, the SUMMIT trial reported that tirzepatide improved quality of life in HFpEF patients and reduced the risk of worsening HF or cardiovascular death [183].

6.2.1.2 Underlying Mechanisms of GLP-1RAs in MASLD and HFpEF Patients

At the hepatic level, GLP-1RAs reduce lipid influx to the liver by increasing insulin secretion and suppressing glucagon release, thereby inhibiting hormone-sensitive lipase activity, decreasing triglyceride hydrolysis, and limiting the release of free fatty acids from adipose tissue [191]. In addition, GLP-1RAs attenuate hepatic lipid accumulation through activation of the AMP-activated protein kinase (AMPK) pathway, which promotes fatty acid β-oxidation and suppresses *de novo* lipogenesis [192,193]. Concomitantly, these agents inhibit NF-κB signaling and the release of pro-inflammatory cytokines and mitigate hepatocellular injury by modulating apoptotic pathways [192,193].

At the cardiac level, GLP-1RAs exert marked cardioprotective effects through both direct myocardial actions and broad systemic metabolic improvements. They reduce myocardial fibrosis, improve cardiac energy metabolism, and increase myocardial glucose utilization, thereby promoting favorable left ventricular remodeling [194,195,196]. Moreover, GLP-1RAs increase NO bioavailability, attenuate endothelial inflammation, and regulate macrophage polarization, leading to improved endothelial function and reduced oxidative stress [197]. In addition to their direct cardiac effects, GLP-1RAs induce weight loss by suppressing appetite and increasing energy expenditure, which in turn improves insulin sensitivity, lowers blood pressure, and alleviates systemic inflammation, indirectly supporting cardiovascular health by restoring metabolic homeostasis.

#### 6.2.2 SGLT2i

6.2.2.1 Clinical Evidence for SGLT2i in MASLD and HFpEF Patients

SGLT2i are a class of glucose-lowering agents that promote weight loss and exert multiple pleiotropic effects. A growing body of clinical evidence highlights the beneficial effects of SGLT2i in patients with MASLD complicated by T2DM. In RCTs using changes in MRI-assessed liver triglyceride contents as the primary endpoint, SGLT2is have been shown to significantly reduce hepatic steatosis [198,199,200]. In the EMPA-REG OUTCOME program, which pooled data from four placebo-controlled trials and one empagliflozin-versus-glimepiride trial that enrolled a total of 2477 patients, empagliflozin significantly reduced ALT levels in patients with T2DM [201]. Furthermore, a meta-analysis including 10 RCTs with 573 T2DM patients with MASLD demonstrated that, compared with other glucose-lowering agents used in these trials, SGLT2i were superior in improving serum ALT levels, attenuating liver fibrosis, and reducing hepatic fat content and body weight [184]. Nevertheless, RCTs specifically designed for patients with MASLD are lacking.

The 2022 ACC/AHA/HFSA HF management guidelines recommend SGLT2is for the treatment of HF patients across the entire spectrum of the left ventricular ejection fraction [133]. The EMPEROR-Preserved and DELIVER trials provided pivotal evidence supporting the prognostic benefits of SGLT2is in patients with HFpEF. In the EMPEROR-Preserved trial, compared with placebo, empagliflozin significantly reduced the risk of the composite endpoint of cardiovascular death or hospitalization for HF in patients with HFpEF [185]. Similarly, the DELIVER trial revealed that dapagliflozin significantly decreased the incidence of the composite outcome of worsening HF or cardiovascular death and reduced the number of hospitalizations for HF in patients with HFpEF [186]. SGLT2i, therefore, are particularly promising therapeutic option for patients with HFpEF complicated by MASLD; however, dedicated studies focusing on this specific population are still urgently needed.

6.2.2.2 Underlying Mechanisms of SGLT2i in MASLD and HFpEF Patients

In the liver, SGLT2i improves MASLD through multiple complementary mechanisms. These agents suppress hepatic de novo lipogenesis, ameliorate IR, modulate lipid metabolism, and increase fatty acid β-oxidation, thereby reducing intrahepatic lipid accumulation [202]. SGLT2i also exerts anti-inflammatory, antioxidant, and antifibrotic effects, including inhibition of proinflammatory cytokine expression via multiple signaling pathways [203,204,205], attenuation of oxidative stress and apoptosis [205,206], and downregulation of profibrotic mediators [202,205]. In addition, by lowering circulating glucose and insulin levels and modulating glucagon secretion, SGLT2i synergistically reduce hepatic lipid synthesis and deposition [207,208].

In the heart, the cardioprotective effects of SGLT2is on cardiac structure and function are mediated primarily through improvements in systemic hemodynamics and metabolic efficiency. By inducing natriuresis and osmotic diuresis, SGLT2is reduce plasma volume, leading to lower blood pressure and improved cardiac preload and afterload [209]. Moreover, SGLT2is have been shown to increase myocardial utilization of ketone bodies and fatty acids, improve mitochondrial energy metabolism, promote more efficient substrate use, and reduce lactate accumulation and oxidative stress. Collectively, these effects alleviate myocardial stress and provide systemic support for functional improvement in HFpEF patients [210].

### 6.3 Unmet Therapeutic Needs and Future Directions

Targeting the shared metabolic pathways underlying MASLD and HFpEF, GLP-1RAs and SGLT2i have demonstrated potential dual-organ benefits. However, the current evidence is largely derived from studies in populations with obesity or T2DM, and the underlying mechanisms remain incompletely elucidated, with insufficient clinical stratification. Whether improvements in hepatic parameters translate into superior cardiac outcomes, and which MASLD phenotypes derive the greatest therapeutic benefit, remain unresolved questions. In addition, there is a lack of integrated diagnostic scores capable of simultaneously capturing hepatic and cardiac dysfunction; the development of such tools could substantially enhance risk stratification.

Future research should adopt a systems-level perspective, interrogating HFpEF and MASLD through the lens of shared metabolic and inflammatory pathways, and integrating liver–heart biological features to refine risk stratification, enable earlier detection, and improve therapeutic targeting. Within this framework, synergistic hepato-cardiac phenotyping and integrated clinical algorithms may provide a biology-informed basis for the co-management of HFpEF and MASLD, offering a practical opportunity to redefine the prevention, diagnosis, and treatment of these increasingly co-prevalent and interrelated syndromes.

## 7. Conclusion

HFpEF has emerged as the predominant form of HF in parallel with the global rise in metabolic dysfunction, yet its pathophysiology remains incompletely understood by conventional cardiac-centered paradigms. By integrating epidemiological and mechanistic evidence, this review highlights MASLD as a central systemic contributor linking metabolic derangements to the myocardial, vascular, and extracardiac abnormalities characteristic of HFpEF patients. In addition, this review highlights the need for hepato-cardiac risk stratification and phenotype-oriented management. Future priorities include validated cross-organ risk models, biomarker-guided treatment, and dedicated trials targeting MASLD-associated HFpEF to enable precise, mechanism-based care.

## Abbreviations

MASLD, metabolic dysfunction–associated steatotic liver disease; T2DM, type 2 diabetes mellitus; CVD, cardiovascular disease; HFpEF, heart failure with preserved ejection fraction; LVEF, left ventricular ejection fraction; HFmrEF, heart failure with mildly reduced ejection fraction; HFrEF, heart failure with reduced ejection fraction; HF, heart failure; aHR, adjusted hazard ratio; CI, confidence interval; SLD, steatotic liver disease; sHR, subdistribution hazard ratio; DLF, disproportionate liver fat; aOR, adjusted odds ratio; IR, insulin resistance; MASH, metabolic dysfunction–associated steatohepatitis; TNF-α, tumor necrosis factor-α; CRP, c-reactive protein; IL, interleukin; EAT, epicardial adipose tissue; VLDL, very-low-density lipoproteins; sdLDL, small dense low-density lipoproteins; FFA, free fatty acid; ATP, adenosine triphosphate; PI3K/PKB, phosphoinositide 3-kinase/protein kinase B; NO, nitric oxide; JNK, c-jun n-terminal kinase; NF-κB, nuclear factor kappa-b; NLRP3, nucleotide-binding domain, leucine-rich-containing family, pyrin domain-containing 3; eNOS, endothelial nitric oxide synthase; sGC, soluble guanylate cyclase; cGMP, cyclic guanosine monophosphate; PKG, protein kinase G; VCAM-1, vascular cell adhesion molecule-1; ICAM-1, intercellular adhesion molecule-1; FGF21, fibroblast growth factor 21; SAA, serum amyloid A; TLR4, toll-like receptor 4; LPS, lipopolysaccharide; TMAO, trimethylamine n-oxide; SCFAs, short-chain fatty acids; BAs, bile acids; TGF-β, transforming growth factor-β; GPR41, g protein-coupled receptors 41; GPR43, g protein-coupled receptors 43; FXR, farnesoid x receptor; TGR5, g protein-coupled bile acid receptor; MRI, magnetic resonance imaging; CMR, cardiac magnetic resonance; PDFF, proton density fat fraction; FIB-4, fibrosis-4 index; APRI, aspartate aminotransferase-to-platelet ratio index; NFS, nafld fibrosis score; RCT, randomized controlled trial; DASH, dietary approaches to stop hypertension; ALT, alanine aminotransferase; AST, aspartate aminotransferase; MedDiet, mediterranean diet; BMI, body mass index; SGLT2i, sodium–glucose cotransporter 2 inhibitors; GLP-1RAs, glucagon-like peptide-1 receptor agonists; GIP, gastric inhibitory polypeptide; AMPK, amp-activated protein kinase.
